# Influence of chronic kidney disease and its severity on the efficacy of semaglutide in type 2 diabetes patients: a multicenter real-world study

**DOI:** 10.3389/fendo.2023.1240279

**Published:** 2023-10-24

**Authors:** María Dolores García de Lucas, Irene Caballero, José Carlos Fernández-García, Manuel Domínguez-Rodríguez, Paloma Moreno-Moreno, Anabel Jiménez-Millán, Manuel Botana-López, Beatriz Avilés, Juan Francisco Merino-Torres, Alfonso Soto, Cristina Tejera, Cristóbal Morales

**Affiliations:** ^1^ Internal Medicine Department, Hospital Costa del Sol, Marbella, Málaga, Spain; ^2^ Endocrinology and Nutrition Department, Virgen Macarena University Hospital, Sevilla, Spain; ^3^ Endocrinology and Nutrition Department, Regional University Hospital, Málaga, Spain; ^4^ Endocrinology and Nutrition Department, Reina Sofía University Hospital, Córdoba, Spain; ^5^ Endocrinology and Nutrition Department, University Hospital, Puerto Real, Cádiz, Spain; ^6^ Endocrinology and Nutrition Department, Lucus Augusti University Hospital, Lugo, Spain; ^7^ Nephrology Department, Regional University Hospital, Málaga, Spain; ^8^ Endocrinology and Nutrition Department, La Fe University Hospital, València, Spain; ^9^ Endocrinology and Nutrition Department, A Coruña University Hospital Complex, A Coruña, Spain; ^10^ Endocrinology and Nutrition Department, Hospital Básico da Defensa, Ferrol, A Coruña, Spain

**Keywords:** type 2 diabetes, chronic kidney disease, GLP-1 receptor agonists, semaglutide, glycosylated hemoglobin, weight loss, estimated glomerular filtration rate, urinary albumin-to-creatinine ratio

## Abstract

**Objectives:**

Semaglutide is a glucagon-like peptide 1 receptor agonist that improves glycemic control and achieves weight loss in type 2 diabetes (T2D) patients. Subcutaneous (s.c.) semaglutide at 1 mg once weekly (OW) is safe in T2D patients with chronic kidney disease (CKD). Whether or not CKD and its severity influence treatment response remains undetermined.

**Method:**

This is an observational, ambispective, multicenter, nationwide, real-world study designed to compare safety/efficacy of OW s.c. 1 mg semaglutide in T2D patients with or without CKD. The influence of CKD severity was also addressed. Patients were followed up for 12 months. Primary end-points were glycosylated hemoglobin (HbA1c), weight, and renal outcomes. Secondary end-points included insulin resistance, atherogenic and hepatic steatosis indexes, and changes in antihyperglycemic medications.

**Results:**

A total of 296 and 190 T2D patients without or with CKD, respectively, were recruited. Baseline CKD risk was moderate, high, or very high in 82, 53, and 45 patients, respectively. Treatment reduced HbA1c by 0.90%–1.20%. Relevant differences were seen neither between non-CKD and CKD patients nor among CKD subgroups. Notable weight losses were achieved in both non-CKD and CKD patients. The median reduction was higher in the former at 6 months (5.90 kg *vs.* 4.50 kg, *P* = 0.008) and at end of study (6.90 kg *vs.* 5.00 kg, *P* = 0.087). A trend toward slightly lower weight losses as CKD severity increased was observed. CKD markers improved across all CKD subgroups. Relevant differences were not observed for other variables, either between non-CKD and CKD patients, or among CKD subgroups. Safety concerns were not reported.

**Conclusion:**

The safety/efficacy of OW s.c. semaglutide to improve glycemic control and weight in T2D patients with CKD is not notably lower than that in T2D patients without renal failure. CKD severity barely influences treatment response. OW s.c. semaglutide can be useful to manage T2D patients with CKD in daily clinical practice.

## Introduction

Glucagon-like peptide 1 receptor agonists (GLP-1 RAs) are incretin mimetics that act through pancreatic mechanisms to exert glucose-lowering effects. Their additional extra-pancreatic mechanisms influence central control of food intake and induce a delay in gastric emptying, thus enabling weight loss through a satiating effect (1). GLP-1 RAs also promote cardiovascular benefits (2–5), by mechanisms that have not yet been fully identified but that may involve, among others, actions on blood pressure and lipid profile (6). This range of actions has positioned GLP-1 RAs above other medications to treat type 2 diabetes (T2D), and as the first-line treatment when either therapy enhancement with an injectable agent is required or the patient is at high cardiovascular risk (CVR) (7, 8).

Semaglutide is a long-acting GLP-1 RA available to be administered by either subcutaneous (s.c.) or oral route. Its structural modifications make it less susceptible to dipeptidyl peptidase‐4 degradation and improve its affinity to albumin, delaying plasma degradation and decreasing renal clearance (9, 10). These hallmarks allow once-weekly (OW) s.c. administration. In the clinical trials of the SUSTAIN program, OW s.c. semaglutide consistently demonstrated superior glycemic control and weight loss versus either other anti-hyperglycemic agents or other GLP-1 RAs, in a variety of T2D patient cohorts [reviewed in (11)]. Increasing reported evidence suggests that, in real-world scenarios, OW s.c. semaglutide mimics the results observed with T2D patients in controlled trials [12–17, reviewed in (18)].

T2D is the first cause of chronic kidney disease (CKD) (19). Renal outcomes such as reduction of albuminuria and delayed progression of diabetic kidney disease (DKD) have been described upon chronic GLP-1 RA treatment (20), and these further reduce cardiovascular death risk (21). The *post-hoc* analyses of the SUSTAIN program found marked reductions in the urinary albumin-to-creatinine ratio (UACR) in T2D patients treated with semaglutide for up to 2 years (22). In the SUSTAIN trials, the kidney-protective effects exerted by OW s.c. semaglutide were more pronounced in patients with preexisting chronic kidney disease (23). The efficacy of OW s.c. semaglutide, in terms of metabolic control and body weight loss, has been barely addressed in T2D patients with established CKD in real-world scenarios. We recently showed that OW s.c. semaglutide safely and significantly improved glycemic control, decreased weight, and ameliorated renal dysfunction after 12 months of treatment of T2D patients with CKD at high risk of progression (24). Since urine is the primary route of excretion of semaglutide (10), we now hypothesize that kidney damage, and its severity, may influence the efficacy of the treatment. Against this background, we recruited a cohort of OW s.c. semaglutide-treated patients with T2D and CKD from several Spanish hospitals. Firstly, we grouped them according to whether or not they had been diagnosed with CKD. Secondly, we grouped CKD patients according to KDIGO severity criteria (19). The aim was to find out if CKD, and its severity, conditions the actions of OW s.c. semaglutide on glycosylated hemoglobin (HbA1c) levels, body weight, and renal and other outcomes, taking T2D patients with no CKD and treated with OW s.c. semaglutide as the reference group.

## Materials and methods

### Study

This was a multicenter, observational, ambispective, nationwide study to assess the efficacy and safety of OW s.c. semaglutide after 6 and 12 months of treatment in real clinical practice conditions. The study involved nine Spanish hospitals: Virgen Macarena University Hospital, Sevilla; Costa del Sol Hospital, Marbella; Regional University Hospital, Málaga; Reina Sofía University Hospital, Córdoba; University Hospital, Puerto Real; Lucus Augusti University Hospital, Lugo; La Fe University Hospital, Valencia; A Coruña University Hospital Complex, A Coruña; Hospital Básico da Defensa, Ferrol.

Inclusion criteria were as follows: T2D diagnosis according to American Diabetes Association (ADA) criteria for ≥6 months (25); age 18 years or older, regardless of sex; anti-hyperglycemic therapy for ≥3 months with oral hypoglycemic agents and/or insulin; new prescription of OW s.c. semaglutide, with immediate withdrawal of other GLP-1 RAs if these were being used; availability at baseline of an assessment of HbA1c, weight, and blood pressure; signed informed consent. Exclusion criteria were as follows: previous experience with semaglutide, regardless if its administration was s.c. or oral; a diagnosis of type 1 or gestational diabetes; participation in interventional clinical studies in the 90 days prior to semaglutide prescription or during the follow-up period; CKD at stage 5; any condition precluding fully understanding of informed consent.

OW s.c. semaglutide was administered in prefilled pen injectors. Physicians determined the maintenance dose and, when considered necessary, the dose changes. Data corresponding to those patients who started treatment with OW s.c. semaglutide in the period between June 2019 and June 2021 were retrospectively collected from the electronic medical record systems of the participating hospitals. Variable values corresponding to baseline (i.e., immediately before the first dose of OW s.c. semaglutide was administered) and follow-up visits at 6 and 12 months from baseline were collected. Data anonymization was guaranteed. All patients signed written informed consent. The study was conducted in accordance with the ethical principles of the Declaration of Helsinki and started once the local ethics committees had approved the study protocol (ID FIS-SEM-2020-01). These were the Research Ethics Committees of the province of Seville, and each one of the Research Ethics Committees of all participating hospitals (date of the last approval: November 2022).

### Categorization of patients

The CKD status of patients at baseline was determined according to KDIGO guidelines, where a combined variable consisting of eGFR [six categories ranging from normal/high eGFR (G1, ≥90 mL/min/1.73 m^2^) to kidney failure (G5, <15 mL/min/1.73 m^2^)] and UACR status [three categories from normal to mildly increased (A1, <30 mg/g) to severely increased (A3, >300 mg/g)] is considered (**Supplementary Table 1**) (19). Those patients who were at low/no risk of CKD at baseline according to these criteria constituted the low risk/no CKD group. Those who were at moderate, high, or very risk of CKD constituted the CKD group.

### Collected variables

The following data were collected at baseline, and at the 6- and 12-month follow-up visits: anthropometric parameters, namely, weight, body mass index (BMI), waist circumference, and blood pressure; analytical parameters, namely, HbA1c, fasting blood glucose, lipid profile, estimated glomerular filtration rate (eGFR, according to the CKD-EPI equation), and UACR; cardiovascular risk factors and history of cardiovascular or other relevant diseases; risk factors for micro/macrovascular complications; use of anti-hyperglycemic, anti-hypertensive, anti-hyperlipidemic, and/or anticoagulant/antiaggregant therapies. The atherogenic index of plasma [AIP, log(triglycerides/high-density lipoprotein)], hepatic steatosis index [HSI, 8 × (serum alanine aminotransferase to serum aspartate aminotransferase ratio) + BMI (+2, if female; +2, if diabetes mellitus)], triglyceride-glucose index [TyG index, Ln(fasting triglycerides × fasting plasma glucose/2)], and fibrosis-4 score [FIB-4, age (years) × aspartate aminotransferase (U/L)/[platelets (10^9^/L) × alanine aminotransferase^1/2^ (U/L)] were calculated.

### End points

The main outcome variables were HbA1c and weight. Their evolution with respect to baseline values and the extent of improvement according to having or not having CKD, as well as according to CKD severity, were analyzed. Combined goals consisting of achieving predefined simultaneous decreases in HbA1c levels and body weight losses were also evaluated. The evolution of CKD markers was also extensively studied.

Secondary outcome variables included indexes of insulin resistance, atherosclerosis-dependent cardiovascular risk, and hepatic steatosis, as well as anti-hyperglycemic medications at baseline and 12 months after OW s.c. semaglutide initiation.

Hypoglycemic episodes, defined according to the ADA criteria (25), other adverse events (AEs), and treatment withdrawal due to gastrointestinal AEs (GI AEs) or any other cause were reported to assess treatment safety.

### Statistical methods

For analysis purposes, groups were formed according to KDIGO CKD risk criteria (see above) in order to perform comparisons and were as follows: no/low CKD risk group; CKD group (encompassing patients categorized as being of moderate, high, or very high CKD risk). The CKD group was further stratified into three subgroups: moderate CKD risk; high CKD risk; very high CKD risk. Analyses were performed using the intended-to-treat (ITT) population. Intra-group analyses were performed using either the paired t test or the Wilcoxon matched-pair signed rank test according to the parametric or non-parametric distribution of variables. Increments between time points were calculated within each group for some variables, and these increments were compared: on the one hand, between the no/low CKD risk group and the CKD group and, on the other hand, within the CKD group, between the moderate CKD risk subgroup, and either the high CKD risk or the very high CKD risk subgroup. These comparisons were performed by using the Mann–Whitney U test. The Spearman’s rho test was used to study the correlation between variables at defined time points. The Fisher’s exact test was used to compare between groups the proportion of patients who achieved defined goals at defined time points, or to compare intra-group the proportion of patients who achieved defined goals between two time points. All statistical tests were performed by using GraphPad Prism 5.0 software (GraphPad, Dotmatics, Bishop’s Stortford, United Kingdom).

## Results

### Baseline hallmarks

**Figure 1:**
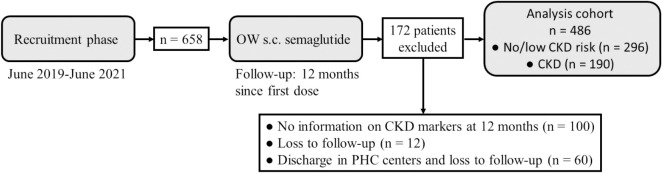
Flowchart diagram of the study. CDK, chronic kidney disease; OW, once weekly; PHC, Primary Health Care; s.c., subcutaneous.

**Figure 1** shows the flowchart diagram of the study. A total of 486 patients were followed for at least 12 months after OW s.c. semaglutide start and were therefore finally recruited. They were grouped according to being at no/low CKD risk (n = 296) or being diagnosed with CKD (n = 190). **Table 1** shows the main baseline features of these groups. Mean age was older in the CKD cohort, which had significantly more male patients. Time since T2D diagnosis was longer in the CKD group. Variable comparison between both groups revealed poorer glycemic control and a worse lipid profile and atherogenic index among CKD patients. These had also a significantly higher FIB-4 index, although the number of CKD patients with established hepatic fibrosis was <4%. Signs of hepatic steatosis were found in almost all patients in both groups. Differences were observed in neither body weight nor waist circumference. CKD patients had suffered more cardiovascular events, either ischemic or non-ischemic, and had a higher use of anti-hypertensive, anti-hyperlipidemic, and hemostatic medications. The number of basal insulin-treated patients was also higher in this group, as was the dose used.

**Table 1:** Baseline characteristics of patients according to being or not being diagnosed with CKD.

Baseline variables	No/low CKD risk (n = 296)	CKD (n = 190)	*P* value
Age, years	58.4 (10.7)	66.6 (10.1)	<0.001
Sex, female, n (%)	157 (53.0)	80 (42.1)	0.020
Time since T2D DG, years, median (IQR)	8.0 (3.0, 14.0)	12.0 (7.0, 20.0)	<0.001
HbA1c, %	8.30 (1.90)	8.75 (1.79)	0.010
Fasting glucose, mg/dL, median (IQR)	147.0 (116.0, 184.0)	158.5 (126.5, 211.5)	0.026
TyG index	9.41 (0.71)	9.62 (0.67)	0.001
TyG index >8.8, n (%)	235 (80.8)	165 (89.7)	0.010
Body weight, kg	98.9 (19.3)	98.5 (16.7)	0.808
BMI, kg/m^2^	36.5 (6.5)	36.4 (5.8)	0.853
Waist circumference, cm	116.9 (12.9)	120.2 (15.1)	0.232
eGFR, mL/min/1.73 m^2^	89.79 (14.37)	49.5 (38.0, 84.0)	<0.001
UACR, mg/g, median (IQR)	6.00 (4.62, 10.00)	58.00 (10.00, 175.00)	<0.001
Creatinine, mg/dL, median (IQR)	0.80 (0.70, 0.90)	1.20 (0.90, 1.60)	<0.001
Cholesterol, mg/dL	172.6 (40.1)	165.5 (39.7)	0.060
LDL, mg/dL	97.5 (35.7)	86.2 (31.1)	<0.001
HDL, mg/dL	43.7 (10.1)	40.3 (9.8)	<0.001
Triglycerides, mg/dL, median (IQR)	172.5 (114.3, 216.8)	182.0 (138.8, 240.8)	0.009
SBP, mm Hg	132.0 (16.3)	134.5 (15.9)	0.126
DBP, mm Hg	77.1 (9.8)	76.6 (10.4)	0.594
AIP, median (IQR), median (IQR)	0.61 (0.42, 0.74)	0.67 (0.52, 0.81)	<0.001
AIP >0.24, n (%)	259 (90.9)	172 (94.5)	0.212
HSI	49.27 (7.53)	48.94 (7.46)	0.669
HSI ≥36, n (%)	234 (99.6)	153 (98.7)	0.566
FIB-4, median (IQR)	1.07 (0.79, 1.36)	1.25 (0.93, 1.59)	0.002
FIB-4 >2.67, n (%)	6 (2.6)	5 (3.3)	0.759
Previous history of			
CHD, n (%)	42 (14.2)	50 (26.3)	0.001
CVD, n (%)	4 (1.3)	16 (8.4)	<0.001
PAD, n (%)	15 (5.1)	32 (16.8)	<0.001
HF, n (%)	10 (3.4)	26 (13.8)	<0.001
NAFLD, n (%)	54 (18.4)	36 (19.1)	0.905
Hypoglycemic medications			
Metformin, n (%)	256 (86.5)	126 (66.3)	<0.001
Glitazones, n (%)	7 (2.4)	3 (1.6)	0.747
DPP-4 inhibitors, n (%)	61 (20.7)	58 (30.5)	0.017
iSGLT2, n (%)	138 (46.6)	100 (52.6)	0.227
Sulfonylureas, n (%)	33 (11.2)	11 (5.8)	0.052
GLP-1 RAs*	113 (38.3)	64 (33.7)	0.334
Insulin, long-acting, n (%)	126 (42.6)	133 (70.0)	<0.001
Insulin, rapid-acting, n (%)	56 (18.9)	71 (38.0)	<0.001
Insulin, long-acting, IU/mL, median (IQR)	36.0 (22.0, 50.0)	44.0 (29.0, 61.0)	0.009
Insulin, rapid-acting, IU/mL, median (IQR)	18.0 (12.0, 30.0)	20.0 (15.2, 30.0)	0.310
Other medications			
ACEI	198 (66.9)	159 (84.6)	<0.001
Beta blockers	60 (20.3)	61 (32.3)	0.004
Alpha blockers	8 (2.7)	17 (9.0)	0.005
Diuretics	93 (31.5)	111 (59.0)	<0.001
CCBs	66 (22.4)	85 (45.0)	<0.001
Statins	191 (64.5)	157 (82.6)	<0.001
PCSK9 inhibitors	1 (0.3)	1 (0.5)	1.000
Fibrates	16 (5.4)	15 (8.0)	0.341
Ezetimibe	32 (10.8)	36 (19.3)	0.011
Anticoagulant drugs	17 (5.8)	21 (11.1)	0.038
Antiaggregant drugs	87 (29.4)	87 (45.8)	<0.001

Data are mean (SD), except where otherwise indicated. CKD diagnosis was made according to KDIGO guidelines (19). There were 10 patients who were classified in the group of high/very high CKD risk because their baseline eGFR values were <45 mL/min/1.73 m^2^, who did not have their baseline UACR assessed, and so they could not be further stratified as being of high or very high CKD risk. There were missed data for several variables, although their proportion was usually insignificant and occurred in >10% of the entire cohort in the following variables only: waist circumference, LDL, SBP, DBP, HSI, FIB-4, and insulin dose, either long-acting and rapid-acting. *Switched to semaglutide immediately after study recruitment. In order to compare the no/low CKD risk group and the CKD group, the two-tailed unpaired t test and the two-tailed Mann–Whitney U test were used for quantitative variables following parametric or non-parametric distribution, respectively. The two-tailed Fisher’s exact test was used to compare qualitative variables.

ACEI, angiotensin-converting enzyme inhibitors; AIP, atherogenic index of plasma; BMI, body mass index; CCBs, calcium channel blockers; CHD, coronary heart disease; CKD, chronic kidney disease; CVD, cerebrovascular disease; DBP, diastolic blood pressure; DG, diagnosis; DPP-4, dipeptidyl peptidase 4; eGFR, estimated glomerular filtration rate; FIB-4, fibrosis-4 score; GLP-1 RAs, glucagon-like peptide-1 receptor agonists; HbA1c, glycosylated hemoglobin; HDL, high-density lipoproteins; HF, heart failure; HSI, hepatic steatosis index; IQR, interquartile range; iSGLT2, sodium-glucose cotransporter 2 inhibitors; LDL, low-density lipoproteins; NAFLD, non-alcoholic fatty liver disease; PAD, peripheral artery disease; PCSK9, proprotein convertase subtilisin/kexin type 9; SBP, systolic blood pressure; SD, standard deviation; TyG index, triglyceride-glucose index; UACR, urine albumin to creatinine ratio.

CKD patients were further stratified according to being at moderate (n = 82), high (n = 53), or very high (n = 45) CKD risk. A total of 10 patients who should be classified as of high or very high CKD risk because their baseline eGFR values were <45 mL/min/1.73 m^2^ did not have their baseline UACR assessed, and so they could not be definitely assigned to one or another subgroup. **Supplementary Table 2** shows the baseline hallmarks of CKD subgroups. There were only slight differences regarding age. A trend toward more years of T2D evolution as CKD risk increases could be envisaged. Apart from the differences in kidney disease markers, no notable differences in baseline variables, history of cardiovascular diseases, or anti-hyperglycemic/other therapies were reported.

### Evolution of glycemic control and body weight

HbA1c levels were significantly reduced at 6 months and continued to improve in the next 6 months in the no/low CKD risk cohort and in CKD patients, regardless of CKD severity (**Table 2**). Body weight also improved significantly in all groups. Weight decrease occurred mainly in the first 6 months, with mean losses in the range of 5–6 kg (**Table 2**). Waist circumference was also significantly reduced in the no/low CKD risk and CKD groups (**Supplementary Table 3**). There were no significant differences in the extent of HbA1c decrease either between no/low CKD risk and CKD patients, or among CKD patients when these were categorized according to severity (**Table 3**). However, the extent of weight loss was significantly different between no/low CKD risk and CKD patients, with a mean loss which was around 2 kg higher in the former after 12 months of OW s.c. semaglutide. In the group of CKD patients, there was a non-statistically significant trend to a more inefficient effect of treatment, in terms of weight loss, in very high risk CKD patients (**Table 3**). The median waist circumference reduction was higher in no/low CKD risk patients at 6 and 12 months, although the difference compared with the CKD group was not statistically significant (**Supplementary Table 4**). It is important to point out that, among CKD patients, those who were administered s.c. semaglutide at 1 mg weekly at least during the 6 last months of follow-up (78.9%) had better results, in terms of HbA1c and body weight decrease, than those who had not reached the full dose at the end of the study (EOS). This difference according to semaglutide dose was not found within the group of no/low CKD risk patients, where 78.2% of them were on the full dose at EOS (**Supplementary Table 5**). On the other hand, the decrease in HbA1c and body weight at EOS in those patients who had already been treated with other GLP-1RAs, although noteworthy, was lower than that reported for those patients naïve to GLP-1RA, whose proportion ranged between 30% and 40% in both no/low CKD risk and CKD cohorts. This finding was observed in both groups (**Supplementary Table 6**).

**Table 2:** HbA1c and body weight over time according to CKD status during 12 months of treatment with semaglutide.

Patients according to CKD status (n = 486)	Baseline	6 months	*P* value*	12 months	*P* value^†^	*P* value^‡^
No/low CKD risk (n = 296)						
HbA1c, %	8.30 (1.90)	6.70 (1.08)	<0.001	6.59 (0.98)	<0.001	0.016
Body weight, kg	98.9 (19.3)	92.7 (18.2)	<0.001	91.5 (16.2)	<0.001	<0.001
CKD (n = 190)						
HbA1c, %	8.75 (1.79)	7.09 (1.14)	<0.001	6.83 (0.91)	<0.001	0.002
Body weight, kg	98.5 (16.7)	93.2 (15.9)	<0.001	92.6 (16.2)	<0.001	<0.001
Moderate risk CKD (n = 82)						
HbA1c, %	8.94 (1.68)	7.12 (1.17)	<0.001	6.90 (0.92)	<0.001	0.075
Body weight, kg	100.4 (17.5)	95.2 (16.6)	<0.001	94.0 (16.0)	<0.001	0.003
High risk CKD (n = 53)						
HbA1c, %	8.34 (1.55)	6.95 (0.97)	<0.001	6.66 (1.01)	<0.001	0.024
Body weight, kg	97.3 (15.4)	90.8 (14.6)	<0.001	92.2 (14.8)	<0.001	0.139
Very high risk CKD (n = 45)						
HbA1c, %	8.68 (1.97)	7.12 (1.26)	<0.001	6.85 (0.76)	<0.001	0.057
Body weight, kg	96.5 (16.9)	92.0 (16.4)	<0.001	92.1 (17.8)	<0.001	0.015

A total of 10 patients who were classified in the group of high/very high CKD risk because their baseline eGFR values were <45 mL/min/1.73 m^2^ did not have their baseline UACR assessed, and so they could not be further stratified as being of high or very high CKD risk. *6 months vs. baseline. ^†^12 months vs. baseline. ^‡^6 months vs. 12 months. Results are expressed as mean (SD). The one-tailed paired t test was used to perform the comparisons.

CKD, chronic kidney disease; HbA1c, glycosylated hemoglobin; SD, standard deviation.

**Table 3:** Extent of change in HbA1c and body weight with respect to baseline at 6 and 12 months after starting semaglutide treatment.

	No/low CKD risk (n = 296)	CKD (n = 190)	*P* value*	Moderate risk (n = 82)	High risk (n = 53)	*P* value^†^	Very high risk (n = 45)	*P* value^‡^
HbA1c, Δ vs. bl (%)								
At 6 months	-1.10 (-2.25, -0.40)	-1.20 (-2.45, -0.50)	0.909	-1.25 (-2.67, -0.50)	-0.80 (-2.00, -0.20)	0.114	-1.25 (-2.30, -0.50)	0.676
At 12 months	-0.90 (-2.10, -0.40)	-1.20 (-2.50, -0.60)	0.133	-1.30 (-2.55, -0.70)	-1.20 (-2.35, -0.05)	0.209	-1.35 (-2.90, -0.60)	0.920
Body weight, Δ vs. bl (kg)								
At 6 months	-5.90 (-9.00, -2.80)	-4.50 (-7.70, -1.60)	0.008	-4.80 (-7.95, -1.67)	-4.50 (-8.90, -2.00)	0.975	-4.15 (-6.30, -1.00)	0.169
At 12 months	-6.90 (-11.15, -2.35)	-5.00 (-9.30, -2.30)	0.087	-6.00 (-10.25, -2.70)	-5.00 (-9.30, -2.25)	0.411	-3.70 (-9.00, -1.85)	0.336

A total of 10 patients who were classified in the group of high/very high CKD risk because their baseline eGFR values were <45 mL/min/1.73 m^2^ did not have their baseline UACR assessed, and so they could not be further stratified as being of high or very high CKD risk. *CKD vs. no/low CKD risk. †High risk CKD vs. moderate risk CKD. ‡Very high risk CKD vs. moderate risk CKD. Results are expressed as median (IQR). The two-tailed Mann–Whitney U test was used to perform the comparisons.

bl, baseline; CKD, chronic kidney disease; HbA1c, glycosylated hemoglobin; IQR, interquartile range; UACR, urine albumin to creatinine ratio.

**Figure 2:**
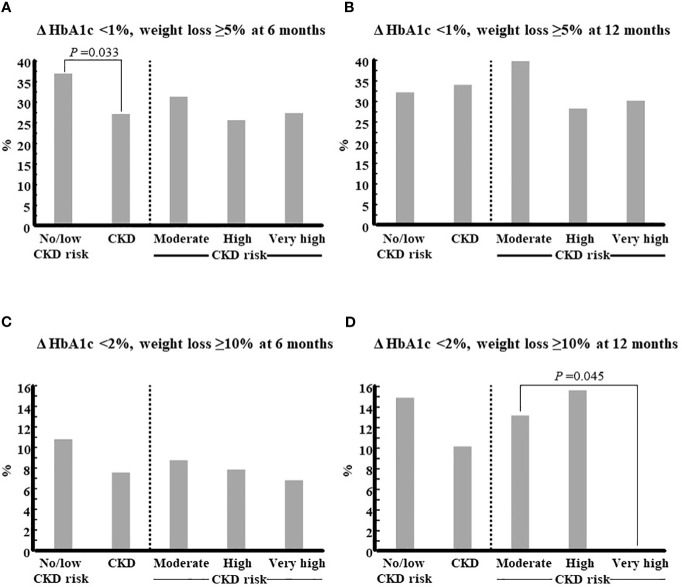
Achievement of combined goals of glycemic control and weight loss according to CKD status. Patients were categorized according to their CKD status at baseline, and the proportion of those who achieved the combined goals indicated in each panel were calculated for each group: **(A)**, D HbA1c <1% and weight loss ≥5% at 6 months; **(B)**, D HbA1c <1% and weight loss ≥5% at 12 months; **(C)**, D HbA1c <2% and weight loss ≥10% at 6 months; **(D)**, D HbA1c <2% and weight loss ≥10% at 12 months. The two-tailed Fisher’s exact test was used to compare, on the one hand, the CKD group with the No/low CKD risk group and, on the other hand, the groups of high or very high CKD risk with the group of moderate CKD risk.CKD, chronic kidney disease; HbA1c, glycosylated hemoglobin. *P <0.05.

After 6 months of treatment, the combined target of HbA1c decrease of at least 1% and loss of at least 5% of body weight was achieved by a proportion of no/low CKD risk patients, which was significantly higher than that of CKD patients (**Figure 2A**), although there were no major differences between both groups after 12 months of treatment (**Figure 2B**). There were no significant differences according to CKD severity, although a trend to a higher proportion of moderate risk CKD patients achieving the combined goal was observed (**Figure 2B**). There were no significant differences in the proportions of no/low CKD risk or CKD patients achieving the more ambitious goal of a decrease of at least 2% in HbA1c and a loss of body weight of 10% or higher (**Figures 2C, D**), although this target was reached by a significantly lower proportion of patients diagnosed with very high CKD risk (**Figure 2D**).

**Figure 3:**
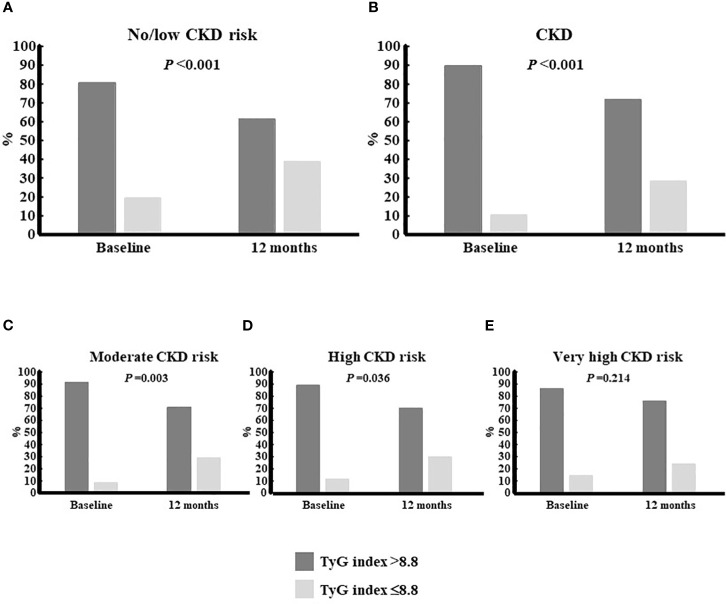
Proportion of patients who achieved a TyG index £8.8 according to CKD status. The cut-off point of 8.8 was used to consider patients as having (above) or not having (equal or below) insulin resistance. Within each group, the proportion of patients above or below the cut-off were reported for the time points corresponding to baseline and 6 months: **(A)**, No/low CKD risk patients; **(B)**, CKD patients; **(C)**, moderate CKD risk patients;**(D)**, high CKD riskpatients; **(E)**, very high CKD risk patients. The one-tailed Fisher’s exact test was used to look for differences between both time points. CKD, chronic kidney disease; TyG index, triglyceride-glucose index.

The TyG index of insulin resistance improved significantly in both cohorts of no/low CKD risk and CKD patients, and in all subgroups of the latter (**Supplementary Table 3**). A slight, non-significant trend to a higher improvement in the CKD cohort (**Supplementary Table 4**), which was irrespective of CKD severity (not shown), could be observed. Nevertheless, although the number of patients who had a TyG index value of ≤8.8 (non-insulin resistance) increased significantly in both the no/low CKD risk and CKD groups after 12 months of treatment (**Figures 3A, B**), when further stratification according to CKD severity was performed, a lower proportion of patients achieving a TyG value ≤8.8 was reported in the subgroup of very high CKD risk (**Figures 3C–E**).

### Anti-hyperglycemic medications

Therapeutic strategies to improve glycemic control had changed remarkably 12 months after the start of the study in both no/low CKD risk and CKD patients (**Supplementary Figure 1**). Before study initiation, the proportion of patients using a GLP-1 RA was lower than 40% in both cohorts. After 12 months of treatment with OW s.c. semaglutide, metformin and, especially, DPP-4 inhibitor use was reduced in both groups whereas, by contrast, the use of iSGLT2 increased. The proportion of patients using basal insulin, which initially was higher in the CKD group, did not change greatly after 12 months, although the dose of basal insulin was reduced, albeit non-significantly: 36.0 (22.0–50.0) IU/mL *vs.* 34.0 (22.0–48.0) IU/mL, median (IQR), in no/low CKD risk patients at baseline and 12 months respectively; 44.0 (29.0–61.0) IU/mL *vs.* 38.0 (24.7–60.0) IU/mL in CKD patients at baseline and 12 months, respectively. The use of rapid insulin significantly decreased in both no/low CKD risk and CKD patients.

### CKD evolution

**Figure 4:**
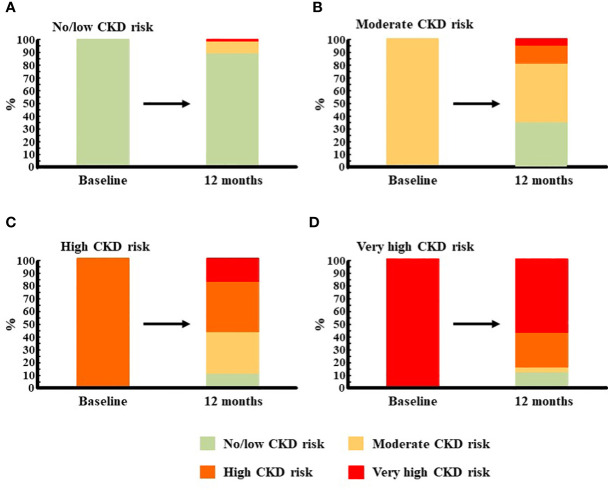
Evolution of CKD risk after 12 months of treatment with semaglutide. The prognosis of CKD was assessed immediately before starting treatment with semaglutide, and 12 months afterwards, according to KDIGO criteria using a variable that combines eGFR (categories G1-G5) and UACR (categories A1-A3) (19). Then, patients were categorized according to their prognosis at baseline, and the proportion of them who had the same prognosis or fell into another category after 12 months of treatment with semaglutide was calculated: **(A)**, No/low CKD risk patients; **(B**), moderate CKD risk patients; **(C)**, high CKD risk patients; **(D)**, very high CKD risk patients. CKD, chronic kidney disease; eGFR, estimated glomerular filtration rate; UACR, urine albumin to creatinine ratio.

In the cohort of CKD patients, no improvement in eGFR after 12 months of treatment, with even a slight decrease in the group of moderate CKD risk, was documented. By contrast, a noticeable improvement in UACR through the 12 months of treatment was observed, especially in the first 6 months (**Table 4**). Such improvement was seen in all CKD subgroups, although it was more pronounced in those with more severe CKD manifestations according to KDIGO criteria (**Table 5**). An improvement in CKD status after 12 months of treatment with OW s.c. semaglutide was reported (**Figure 4**). More than 30% of patients diagnosed with moderate CKD risk at baseline had reached the status of no/low CKD risk at 12 months (**Figure 4B**), and more than 40% of patients initially at high CKD risk were at either moderate or low/no CKD risk at 12 months (**Figure 4C**). Finally, more than 40% of patients initially at very high CKD risk were at high, moderate, or low/no CKD risk at 12 months (**Figure 4D**).

**Table 4:** Renal function variables over time according to CKD status during 12 months of treatment with semaglutide.

Patients according to CKD status (n = 486)	Baseline	6 months	*P* value*	12 months	*P* value^†^	*P* value^‡^
No/low CKD risk (n = 296)						
eGFR, mL/min/1.73 m^2^	90.00 (81.00, 98.00)	90.00 (84.25, 97.00)	0.277	90.00 (82.58, 98.00)	0.313	0.196
UACR, mg/g	6.00 (4.62, 10.00)	6.00 (4.20, 9.00)	0.221	6.00 (4.00, 10.00)	0.384	0.079
CKD (n = 190)						
eGFR, mL/min/1.73 m^2^	49.50 (38.00, 84.00)	50.00 (40.00, 80.00)	0.811	48.00 (39.25, 80.75)	0.020	0.076
UACR, mg/g	58.00 (10.00, 175.00)	38.10 (6.00, 128.00)	<0.001	23.00 (6.10, 118.50)	<0.001	0.688
Moderate risk CKD (n = 82)						
eGFR, mL/min/1.73 m^2^	80.00 (55.00, 98.00)	72.50 (55.00, 92.00)	0.010	78.00 (48.75, 90.00)	<0.001	0.106
UACR, mg/g	48.20 (11.50, 87.90)	27.70 (7.95, 66.85)	<0.001	20.00 (6.90, 52.80)	0.003	0.614
High risk CKD (n = 53)						
eGFR, mL/min/1.73 m^2^	47.00 (40.86, 66.50)	47.00 (40.00, 68.00)	0.703	46.00 (40.00, 66.50)	0.344	0.647
UACR, mg/g	45.00 (6.00, 360.50)	13.50 (6.00, 146.30)	<0.001	22.00 (5.41, 177.90)	0.002	0.717
Very high risk CKD (n = 45)						
eGFR, mL/min/1.73 m^2^	32.00 (20.50, 40.50)	37.00 (28.00, 45.00)	0.111	35.00 (25.75, 43.25)	0.444	0.262
UACR, mg/g	158.50 (46.25, 694.50)	100.00 (31.45, 379.80)	<0.001	55.00 (15.50, 154.00)	<0.001	0.200

A total of 10 patients who were classified in the group of high/very high CKD risk because their baseline eGFR values were <45 mL/min/1.73 m^2^ did not have their baseline UACR assessed, and so they could not be further stratified as being of high or very high CKD risk. *6 months vs. baseline. ^†^12 months vs. baseline. ^‡^6 months vs. 12 months. Results are expressed as median (IQR). The two-tailed Wilcoxon matched-pairs signed-rank test was used to perform the comparisons.

CKD, chronic kidney disease; eGFR, estimated glomerular filtration rate; IQR, interquartile range; UACR, urine albumin to creatinine ratio.

**Table 5:** Extent of change in renal function variables with respect to baseline at 6 and 12 months after starting semaglutide treatment.

	No/low CKD risk (n = 296)	CKD (n = 190)	*P* value*	Moderate risk (n = 82)	High risk (n = 53)	*P* value^†^	Very high risk (n = 45)	*P* value^‡^
eGFR, Δ vs. bl, mL/min/1.73 m^2^								
At 6 months	0.00 (-3.00, 4.00)	0.00 (-5.00, 5.00)	0.306	-1.00 (-9.77, 3.00)	-1.00 (-3.80, 3.72)	0.224	1.00 (-2.00, 7.00)	0.011
	-0.93 (14.97)	1.96 (19.66)		-3.81 (11.19)	0.60 (9.00)		11.91 (33.10)	
At 12 months	0.00 (-5.55, 4.00)	-2.00 (-8.75, 4.00)	0.134	-4.85 (-13.08, 0.25)	-3.00 (-8.00, 6.00)	0.216	0.00 (-5.25, 7.00)	0.006
	-0.39 (11.13)	-0.42 (21.86)		-4.70 (24.48)	-2.01 (10.78)		7.93 (24.39)	
UACR, Δ vs. bl, mg/g								
At 6 months	0.00 (-1.50, 0.00)	-13.40 (-76.45, 0.00)	<0.001	-10.00 (-44.45, 0.00)	-3.00 (-108.00, 0.00)	0.346	-28.50 (-177.00, 0.00)	0.017
	2.14 (20.53)	-71.85 (190.00)		-18.43 (57.21)	-95.34 (201.30)		-135.80 (283.00)	
At 12 months	0.00 (-1.50, 2.00)	-22.80 (-92.50, 0.00)	<0.001	-11.33 (-51.40, 0.00)	-23.00 (-162.20, 0.00)	0.094	-71.00 (-336.40, -9.50)	0.003
	7.05 (39.84)	-85.65 (282.80)		-16.61 (66.58)	-96.59 (404.50)		-191.70 (317.40)	

A total of 10 patients who were classified in the group of high/very high CKD risk because their baseline eGFR values were <45 mL/min/1.73 m^2^ did not have their baseline UACR assessed, and so they could not be further stratified as being of high or very high CKD risk. *CKD vs. no/low CKD risk. †High risk CKD vs. moderate risk CKD. ‡Very high risk CKD vs. moderate risk CKD. Results are expressed as median (IQR), above, and mean (SD), below. The two-tailed Mann–Whitney U test was used to perform the comparisons.

bl, baseline; CKD, chronic kidney disease; eGFR, estimated glomerular filtration rate; IQR, interquartile range; SD, standard deviation; UACR, urine albumin to creatinine ratio.

### Association between severity of kidney disease and primary targets of semaglutide

No correlation was found between UACR and HbA1c after either 6 or 12 months of treatment. A slight although significant correlation could be seen between UACR and body weight at 6 months. However, this was not maintained at 6 months afterward (**Supplementary Table 7**). Furthermore, the extent of UACR change was not associated with the magnitude of weight loss (**Supplementary Table 8**).

### Indexes of atherogenesis and hepatic steatosis

Both AIP and HSI index values decreased significantly in both low/no CKD risk and CKD cohorts after 12 months (**Supplementary Figures 2B, D**). The decrease was not of enough magnitude to have significantly less patients below risk cutoff values (0.24 for AIP, 36 for HSI), although the proportion of those who achieved this goal was higher in the non-CKD group (**Supplementary Figures 2A, C**).

### Safety

Three (1.0%) and four (2.1%) patients in the low/no CKD risk cohort and CKD cohort, respectively, reported at least one severe hypoglycemic episode during the follow-up period. One CKD patient reported several events of urinary tract infection. No other severe AEs, either treatment or non-treatment related, were documented.

In the low/no CKD risk cohort, 31 (10.5%) patients reported moderate (n = 24) or severe (n = 7) GI AEs in the first 6 months of treatment. Thereafter, seven and four patients reported moderate or severe GI AEs, respectively. In the CKD group, 22 (11.6%) patients reported moderate (n = 18) or severe (n = 4) GI AEs in the first half of the follow-up period. Thereafter, four and three patients had moderate or severe GI AEs, respectively. GI AEs caused treatment withdrawal in 13 cases. As anticipated, among those patients who remained on treatment with semaglutide by the EOS, 78.2% and 78.9% of those in the low/no CKD risk cohort and CKD cohort, respectively, were being administered the full dose of 1 mg weekly at that time whereas only one patient remained with the initial dose of 0.25 mg weekly. In the low/no CKD risk cohort, one patient suspended treatment at 7 months due to the absence of effect on body weight. In the CKD cohort, two patients developed intolerance to OW s.c. semaglutide and withdrew treatment. Finally, another CKD patient withdrew from treatment after 4 months for unknown causes.

## Discussion

The SUSTAIN trials revealed that OW s.c. semaglutide was safe for kidney function in T2D patients (22). Furthermore, they showed that this therapy improved CKD markers (23). Nevertheless, the influence of CKD on semaglutide-related glycemic control and weight loss was not systematically addressed. Urine is the primary route of excretion of semaglutide. Its intact form in this fluid accounts for roughly 3% of the administered dose (10). Thus, assessing if CKD influences the efficacy of semaglutide by modifying its availability may be of interest. Evidence describing that OW s.c. semaglutide is safe and efficient to target glycemic control and weight loss in T2D patients in the daily clinical practice is becoming increasingly available [12–17, reviewed in (18)]. We recently showed that OW s.c. semaglutide therapy provided benefits to T2D patients diagnosed with CKD (24). Nevertheless, to the best of our knowledge, this is the first study to compare the efficacy and safety of OW s.c. semaglutide between non-CKD and CKD T2D patients, as well as to assess if this treatment may be influenced by CKD severity.

In our cohort, CKD patients were older, had more years of T2D evolution, and had poorer glycemic control. Their baseline body weight did not differ from that of non-CKD patients, and they had a better lipid profile subsequent to a higher statin use. Many patients in both groups were already using GLP-1 RAs. The switch to OW s.c. semaglutide was due to failure to meet HbA1c or weight targets with previous GLP-1 RAs, need to improve cardiovascular status and/or adherence (OW *vs.* daily), or delaying treatment intensification, as previously described (26). On the other hand, the introduction of GLP-1 RAs involved withdrawal of the incretin-related iDPP4 treatment, and the use of iSGLT2 notably increased because of the well-known effects on renal disease (27).

Semaglutide significantly reduced HbA1c levels and body weight throughout the study in non-CKD patients, as well as in CKD patients regardless of severity, to an extent which was roughly similar to that described in the aforementioned real-world studies (12–18). The effect of treatment was higher during the first 6 months. There were no remarkable differences regarding HbA1c decrease between non-CKD and CKD patients, or among CKD subgroups. The efficacy of OW s.c. semaglutide in reducing body weight was slightly better in non-CKD than in CKD patients. Among the latter, efficacy seemed to be lower as severity increased. This finding, together with the fact that baseline HbA1c values were higher in CKD patients, resulted in a lower proportion of CKD patients achieving the combined target of either ≥5% weight loss and <7.5% HbA1c, or ≥10% weight loss and <7.0% HbA1c, when compared with non-CKD patients. Notably, while the difference in efficacy of OW s.c. semaglutide according to whether or not it was administered at the full dose of 1 mg was not remarkable in the non-CKD patients, those patients with CKD who did not use OW 1 mg as maintenance dose had less benefit from therapy in terms of glycemic control and weight loss. Finally, the finding that the improvement in HbA1c and weight at EOS in those patients previously treated with other GLP-1 RAs was smaller than that observed in GLP-1RA-naïve patients was in line with other real-world studies (18).

The TyG index is considered a surrogate marker of metabolic syndrome (28) and is used to assess insulin resistance. The TyG index decreased to a similar extent in non-CKD patients and in CKD patients as well, regardless of CKD severity. Improvement could be seen already by 6 months after OW s.c. semaglutide was started. On the other hand, waist circumference, a well-known cardiovascular risk factor (29), was reduced by EOS in both non-CKD and CKD patients.

After 12 months of treatment with OW s.c. semaglutide, the diagnosis of CKD patients improved. In the three subgroups, around 30%–40% of patients had a less severe CKD diagnosis at EOS. The results of evolution of eGFR and, especially, UACR, invite us to suggest that the beneficial effect of OW s.c. semaglutide becomes higher as baseline CKD severity increases. These results are in line with those described for CKD patients in SUSTAIN trials (22). On the other hand, while an association between UACR and HbA1c evolution was never seen in our cohorts, there was a direct correlation between UACR and body weight, which suggests that weight loss promotes improvement of albuminuria, as previously described (30, 31). Bariatric surgery was found to reduce the severity of albuminuria 1 year after the procedure, and authors stated that an improvement in albuminuria should be a marker of the resolution of diabesity-mediated renal injury following any successful weight-loss strategy (30). Finally, atherogenic and hepatic steatosis indexes improved in both non-CKD and CKD patients. Benefits of semaglutide regarding cardiovascular outcomes and fatty liver disease have been previously described in T2D patients (3, 32). Moreover, as recently described, GLP-1 RAs are able to induce non-alcoholic steatohepatitis resolution, reduction in weight and fat content, improvements in hepatic injury biomarkers, and non-alcoholic fatty liver disease in a high percentage of T2D patients (33). In our cohort, both indexes decreased regardless of CKD diagnosis. Nevertheless, the number of patients whose index values scored under the risk cutoffs remained high in both groups at EOS.

Twelve-month treatment with OW s.c. semaglutide was safe, in line with previous real-world series (12–18), regardless of CKD diagnosis. Patients were properly trained to minimize GI AE symptoms (34), which were usually transient and caused withdrawal in only 13 patients over the entire cohort, the impact being similar in non-CKD and CKD patients. The proportion of patients who had ≥1 severe hypoglycemic episode during the follow-up period was <2.5% in both non-CKD and CKD cohorts, and no severe treatment-related AEs were documented. Furthermore, the use of OW s.c. semaglutide allowed a reduction in the use of rapid insulin in both cohorts. A trend toward a lower dose of basal insulin, which was still not statistically significant, was also observed at EOS.

Our study has limitations. The influence of therapies other than semaglutide and/or comorbidities on the assessed variables was not considered. Not surprisingly, at baseline, CKD patients were older and had poorer glycemic control than those with no CKD or low CKD risk. When the study started, the use of medications such as statins and diuretics, was not similar in the non-CKD and CKD cohorts. The design of the study has precluded us to analyze the role played by iSGLT2 on the evolution of CKD markers. Nevertheless, probably, it has not been very different in the CKD and non-CKD groups, since the proportion of patients on iSGT2 treatment was not very different before the start and at the end of follow-up. The size of the cohort precluded multivariate analyses, and there was the additional inconvenience that the follow-up period coincided with the advent of the COVID-19 crisis, which hampered proper data gathering and patient surveying. Finally, adherence was not considered either. Nevertheless, we still believe that our cohort conforms with the everyday clinical population, thus providing a reliable picture of the scenario of CKD associated with T2D.

In conclusion, the results presented herein allows us to confirm that OW s.c. semaglutide exerts positive effects on glycemic control, weight loss, and renal function in T2D patients diagnosed with CKD. Those using the full dose will benefit most from the treatment. Weight loss seems to be slightly higher in non-CKD patients and, among those diagnosed with CKD, in those with less severe forms. Otherwise, renal disease and its severity do not seem to preclude either the safety or the efficacy of this therapy. Thus, our nationwide real-world study supports the suitability of OW 1 mg s.c. semaglutide to treat T2D patients with CKD and poor glycemic control and/or obesity/overweight.

## Data availability statement

The original contributions presented in the study are included in the article/**Supplementary Material**. Further inquiries can be directed to the corresponding author.

## Ethics statement

The studies involving human participants were reviewed and approved by CEIM SEVILLE. The patients/participants provided their written informed consent to participate in this study.

## Author contributions

MG, CM, and IC contributed to the conception, design of the work the acquisition, interpretation of data, writing—original draft preparation, writing—review and editing, and supervision. JF-G, MD-R, PM-M, AJ-M, MB-L, JM-T, AS, and CT contributed to the acquisition of data and revised the work. BA contributed to interpretation of data, writing—review and editing, and supervision. MG was a major contributor in interpretation of data, writing—original draft preparation, writing-review and editing, and supervision. All authors read and approved the final manuscript. All authors meet the criteria for authorship stated in the Uniform Requirements for Manuscripts Submitted to Biomedical Journals.
