# Supplementary material for: Influence of chronic kidney disease and its severity on the efficacy of semaglutide in type 2 diabetes patients: a multicenter real-world study
Source: Front Endocrinol (Lausanne). 2023 Oct 24;14:1240279. doi: 10.3389/fendo.2023.1240279 (PMC10634592; doi:10.3389/fendo.2023.1240279)
Supplement: Supplementary file 1 [file Table_1.pdf]

Supplementary Table 1. Prognosis of CKD based on GFR and albuminuria categories according to KDIGO criteria

CKD risk			
**No*/Low**	**Moderate**	**High**	**Very high†**
● UACR <30 mg/g **(A1)**<br>and<br>eGFR ≥60 ml/min/1.73 m^2 **(G1 or G2)**	● UACR <30 mg/g **(A1)**<br>and<br>eGFR 45-59 ml/min/1.73 m^2 **(G3a)**<br><br>● UACR 30-300 mg/g **(A2)**<br>and<br>eGFR ≥60 ml/min/1.73 m^2 **(G1 or G2)**	● UACR <30 mg/g **(A1)**<br>and<br>eGFR 30-44 ml/min/1.73 m^2 **(G3b)**<br><br>● UACR 30-300 mg/g **(A2)**<br>and<br>eGFR 45-59 ml/min/1.73 m^2 **(G3a)**<br><br>● UACR >300 mg/g **(A3)**<br>and<br>eGFR ≥60 ml/min/1.73 m^2 **(G1 or G2)**	● UACR <30 mg/g **(A1)**<br>and<br>eGFR ≤29 ml/min/1.73 m^2 **(G4 or G5)**<br><br>● UACR 30-300 mg/g **(A2)**<br>and<br>eGFR ≤44 ml/min/1.73 m^2 **(G3b, G4 or G5)**<br><br>● UACR >300 mg/g **(A3)**<br>and<br>eGFR ≤59 ml/min/1.73 m^2 **(G3a, G3b, G4 or G5)**

*No CKD if no other markers of kidney disease are found. †Patients with eGFR <15 ml/min/1.73 m^2 at baseline were not included in the study. KDIGO criteria for CKD risk consist of combinations of albuminuria (A1-A3) and eGFR (G1-G5) categories (19).

CKD, chronic kidney disease; eGFR, estimated glomerular filtration rate; KDIGO, Kidney Disease: Improving Global Outcomes; UACR, urine albumin to creatinine ratio.

Supplementary Table 2. Baseline characteristics of CKD patients according to their CKD status

Baseline variables	CKD risk		
	Moderate (n = 82)	High (n = 53)	Very high (n = 45)
Age, years	65.1 (9.7)	67.0 (10.9)	69.3 (9.8)
Sex, female, n (%)	30 (36.6)	22 (41.5)	25 (55.5)
Time since T2D DG, years, median (IQR)	10.5 (5.0, 20.0)	12.0 (9.0, 21.0)	15.0 (10.0, 22.0)
HbA1c, %	8.94 (1.68)	8.34 (1.55)	8.68 (1.97)
Fasting glucose, mg/dL, median (IQR)	160.0 (128.5, 212.5)	159.0 (129.0, 192.5)	155.0 (117.8, 218.5)
TyG index	9.58 (0.61)	9.53 (0.61)	9.64 (0.70)
TyG index >8.8, n (%)	73 (91.2)	47 (88.7)	37 (86.0)
Body weight, kg	100.4 (17.5)	97.3 (15.4)	96.5 (16.9)
BMI, kg/m^2	37.2 (6.2)	35.4 (5.5)	36.2 (5.9)
Waist circumference, cm	122.0 (11.1)	118.5 (10.6)	120.1 (22.7)
eGFR, mL/min/1.73m^2	77.86 (22.50)	54.99 (20.30)	28.7 (14.8)
UACR, mg/g, median (IQR)	48.20 (11.50, 87.90)	45.00 (6.00, 360.50)	158.50 (46.25, 694.50)
Creatinine, mg/dL, median (IQR)	0.90 (0.80, 1.10)	1.30 (1.20, 1.50)	1.70 (1.40, 2.07)
Cholesterol, mg/dL	165.5 (41.7)	167.0 (34.3)	163.1 (45.2)
LDL, mg/dL	87.6 (33.6)	88.5 (25.8)	81.3 (33.2)
HDL, mg/dL	40.0 (9.9)	40.8 (8.7)	40.7 (10.9)
Triglycerides, mg/dL, median (IQR)	169.0 (134.5, 243.0)	178.0 (135.5, 224.5)	184.5 (162.3, 238.8)
AIP, median (IQR)	0.64 (0.49, 0.78)	0.67 (0.51, 0.78)	0.70 (0.58, 0.86)
AIP >0.24, n (%)	75 (94.9)	50 (94.3)	(93.0)
HSI	50.19 (8.23)	47.82 (6.32)	48.39 (7.58)
HSI, n (%)	62 (96.9)	46 (100)	39 (100)
FIB-4, median (IQR)	1.22 (0.90, 1.49)	1.22 (0.85, 1.65)	1.46 (0.80)
FIB-4 >2.67, n (%)	2 (3.2)	0 (0)	2 (5.3)
Previous history of			
CHD, n (%)	22 (26.8)	12 (22.6)	13 (28.9)
CVD, n (%)	5 (6.1)	7 (13.2)	3 (6.7)
PAD, n (%)	12 (14.6)	8 (15.1)	8 (17.8)
HF, n (%)	7 (8.6)	7 (13.2)	8 (17.8)

NAFLD, n (%)	13 (16.0)	16 (30.8)	7 (15.5)
Hypoglycemic medications			
Metformin, n (%)	68 (82.9)	35 (66.0)	18 (40.0)
Glitazones, n (%)	1 (1.2)	2 (3.8)	0 (0)
DPP-4 inhibitors, n (%)	27 (32.9)	15 (28.3)	14 (31.1)
iSGLT2, n (%)	47 (57.3)	30 (56.6)	19 (42.2)
Sulfonylureas, n (%)	9 (11.0)	0 (0)	2 (4.4)
GLP-1 RAs*	24 (29.3)	23 (43.4)	14 (31.1)
Insulin, long-acting, n (%)	56 (68.3)	38 (71.7)	31 (68.9)
Insulin, rapid-acting, n (%)	25 (30.9)	21 (40.4)	19 (43.2)
Insulin, long-acting, IU/mL, median (IQR)	44.0 (33.0, 70.0)	37.0 (25.5, 55.0)	46.0 (28.5, 63.5)
Insulin, rapid-acting, IU/mL, median (IQR)	20.0 (17.5, 30.0)	20.0 (12.0, 26.0)	28.0 (13.5, 32.0)
Other medications			
ACEI	61 (76.2)	48 (90.6)	40 (88.9)
Beta blockers	27 (33.3)	16 (30.2)	14 (31.1)
Alpha blockers	6 (7.4)	6 (11.3)	4 (8.9)
Diuretics	42 (51.8)	33 (63.5)	30 (66.7)
CCBs	32 (39.5)	23 (43.4)	25 (55.6)
Statins	61 (74.4)	46 (86.8)	41 (91.1)
PCSK9 inhibitors	0 (0)	1 (2.0)	0 (0)
Fibrates	6 (7.3)	4 (7.7)	2 (4.5)
Ezetimibe	8 (9.9)	14 (27.4)	10 (22.7)
Anticoagulant drugs	4 (4.9)	7 (13.5)	9 (20.0)
Antiaggregant drugs	34 (41.5)	29 (54.7)	17 (37.8)

Data are mean (SD), except otherwise indicated. CKD risk was determined according to KDIGO guidelines, which are based on eGFR and UACR values (19). *Switched to semaglutide immediately after study recruitment.

ACEI, angiotensin converting enzyme inhibitors; AIP, atherogenic index of plasma; BMI, body mass index; CCBs, calcium channel blockers; CHD, coronary heart disease; CKD, chronic kidney disease; CVD, cerebrovascular disease; DBP, diastolic blood pressure; DPP-4, dipeptidyl peptidase 4; eGFR, estimated glomerular filtration rate; FIB-4, fibrosis-4 score; GLP-1 RAs, Glucagon-like peptide-1 receptor agonists; HbA1c, glycosylated hemoglobin; HDL, high-density lipoproteins; HF, heart failure; HSI, hepatic steatosis index; IQR, interquartile range; iSGLT2, sodium-glucose cotransporter 2 inhibitors; LDL, low-density lipoproteins; NAFLD, non-alcoholic fatty liver disease; PAD, peripheral artery disease; PCSK9, proprotein convertase subtilisin/kexin type 9; SBP, systolic blood pressure; SD, standard deviation; TyG index, triglyceride-glucose index; UACR, urine albumin to creatinine ratio.

Supplementary Table 3. Waist circumference and TyG index over time according to CKD status during 12 months of treatment with semaglutide

Patients according to CKD status (n = 486)	Baseline	6 months	*P* value*	12 months	*P* value†	*P* value‡
No/low CKD risk (n = 296)						
Waist circumference (cm)	116.9 (12.9)	109.6 (10.5)	<0.001	108.9 (10.3)	<0.001	0.003
TyG index	9.41 (0.71)	9.01 (0.58)	<0.001	8.99 (0.56)	<0.001	0.231
CKD (n = 190)						
Waist circumference (cm)	120.2 (15.1)	112.3 (13.1)	<0.001	109.1 (12.0)	<0.001	<0.001
TyG index	9.62 (0.67)	9.23 (0.59)	<0.001	9.06 (0.62)	<0.001	0.002
Moderate risk CKD (n = 82)						
Waist circumference (cm)	122.0 (11.1)	114.6 (5.8)	0.049	113.1 (7.6)	0.125	0.125
TyG index	9.58 (0.61)	9.19 (0.54)	<0.001	9.05 (0.66)	<0.001	0.092
High risk CKD (n = 53)						
Waist circumference (cm)	118.5 (10.6)	109.7 (7.8)	0.001	106.7 (7.9)	0.004	0.004
TyG index	9.53 (0.61)	9.21 (0.68)	<0.001	8.99 (0.69)	0.015	0.076
Very high risk CKD (n = 45)						
Waist circumference (cm)	120.1 (22.7)	113.9 (22.7)	0.198	109.1 (19.4)	0.031	0.156
TyG index	9.64 (0.70)	9.29 (0.57)	0.001	9.12 (0.45)	<0.001	0.005

Ten patients who were classified in the group of high/very high CKD risk because their baseline eGFR values were <45 mL/min/1.73m^2, did not have their baseline UACR assessed, andn so they could not be further stratified as being of high or very high CKD risk. *6 months *vs.* baseline. †12 months *vs.* baseline. ‡6 months *vs.* 12 months. Results are expressed as mean (SD). The one-tailed paired t test or the one-tailed Wilcoxon matched-pairs signed rank test were used to perform the comparisons.

CKD, chronic kidney disease; HbA1c, glycosylated hemoglobin; SD, standard deviation; TyG index, triglyceride-glucose index; UACR, urine albumin to creatinine ratio.

Supplementary Table 4. Extent of change in waist circumference and TyG index with respect to baseline at 6 and 12 months after starting semaglutide treatment

Variables	No/low CKD risk (n = 296)	CKD (n = 190)	*P* value*
Waist circumference, Δ *vs*. bl, cm			
At 6 months	-7.5 (-10.2, -2.0)	-5.0 (-8.0, 0.0)	0.076
At 12 months	-11.0 (-18.0, -2.0)	-8.0 (-12.0, -2.0)	0.197
TyG index , Δ *vs*. bl			
At 6 months	-0.34 (-0.75, 0.01)	-0.42 (-0.80, 0.05)	0.924
At 12 months	-0.25 (-0.68, 0.10)	-0.38 (-0.81, 0.02)	0.175

*CKD *vs.* No/low CKD risk. Results are expressed as median (IQR). The two-tailed Mann-Whitney U test was used to perform the comparisons.

bl, baseline; CKD, chronic kidney disease; HbA1c, glycosylated hemoglobin; IQR, interquartile range; TyG index, triglyceride-glucose index; UACR, urine albumin to creatinine ratio.

Supplementary Table 5. HbA1c decrease and body weight loss at 12 months according to semaglutide dose

Variables	Semaglutide, OW s.c. dose		
	1 mg ≥6 months until EOS	<1 mg until EOS*	*P* value
HbA1c, Δ *vs*. bl (%)			
No/low CKD risk group	-1.00 (-2.45, -0.50)	-1.25 (-3.75, -0.60)	0.277
CKD group	-1.40 (-2.65, -0.77)	-1.05 (-2.35, -0.30)	0.075
Body weight, Δ *vs*. bl (kg)			
No/low CKD risk group	-7.00 (-12.38, -3.10)	-6.95 (-12.00, -1.45)	0.198
CKD group	-7.00 (-11.05, -3.00)	-4.45 (-7.37, -3.00)	0.040

Within both No/low CKD risk and CKD cohorts, patients were grouped according to having used either the full dose of semaglutide of 1 mg weekly at least during the last 6 months of the follow-up period, or not having reached the 1 mg dose by the EOS. Subsequently, the extent of HbA1c decrease and weight loss was calculated for each subgroup. *All patients were being administered OW 0.5 mg, except one patient in the No/low CKD risk group, who was using the 0.25 mg OW dose. Results are median (IQR). The one-tailed Mann-Whitney U test was used to perform the comparisons.

CKD, chronic kidney disease; EOS, end of study; HbA1c, glycosylated hemoglobin; IQR, interquartile range; OW, once weekly; s.c., subcutaneous.

Supplementary Table 6. HbA1c decrease and body weight loss at 12 months according to previous experience with GLP-1 RAs

Variables	Patients naïve to GLP-1 RA	Patients switching from another GLP-1 RA	*P* value
HbA1c, Δ *vs.* bl (%)			
No/low CKD risk group	-1.50 (-4.50, -0.70)	-0.60 (-1.10, 0)	<0.001
CKD group	-1.65 (-2.95, -0.90)	-0.75 (-1.47, -0.05)	<0.001
Body weight, Δ *vs.* bl (kg)			
No/low CKD risk group	-9.50 (-13.00, -3.70)	-5.00 (-8.00, -2.00)	<0.001
CKD group	-6.25 (-10.78, -2.30)	-4.90 (-7.40, -2.15)	0.071

Within both No/low CKD risk and CKD cohorts, patients were grouped according to having or not having used other GLP-1 RAs before this study (38.3% and 33.7% of patients in the No/low CKD risk group and CKD group, respectively, switched to OW s.c. semaglutide from other GLP-1 RAs). Subsequently, the extent of HbA1c decrease and weight loss was calculated for each subgroup. Results are median (IQR). The one-tailed Mann-Whitney U test was used to perform the comparisons.

CKD, chronic kidney disease; GLP-1 RAs, Glucagon-like peptide-1 receptor agonists; HbA1c, glycosylated hemoglobin; IQR, interquartile range; OW, once weekly; s.c., subcutaneous.

Supplementary Table 7. Correlation between UACR and weight loss or glycemic control in patients diagnosed with CKD at baseline (n = 190)

HbA1c (Δ *vs*. bl) vs. UACR (Δ *vs*. bl)	6 months	12 months
Spearman´s Rho	0.005	-0.018
P value	0.476	0.431
**Body weight (Δ *vs*. bl) vs. UACR (Δ *vs*. bl)**	**6 months**	**12 months**
Spearman´s Rho	0.144	0.071
P value	0.044	0.245

Patients diagnosed with either moderate, high or very high risk of CKD at baseline were grouped, and the correlation between their changes in UACR and either HbA1c or body weight was calculated at 6 and 12 months after the start of semaglutide treatment. The one-tailed Spearman´s Rho test was used.

bl, baseline; CKD, chronic kidney disease; HbA1c, glycosylated hemoglobin; UACR, urine albumin to creatinine ratio.

Supplementary Table 8. Change in UACR according to the magnitude of weight loss in patients diagnosed with CKD at baseline

Weight loss (Δ *vs.* bl)	At 6 months	*P* value*	At 12 months	*P* value†
<1	-3.00 (-70.00, 0.00)		-10.00 (-90.00, 2.00)	
1-<5	-11.00 (-65.50, 0.00)	0.270	-22.00 (-104.20, 0.00)	0.240
5-<10	-13.40 (-72.00, 0.00	0.310	-23.00 (-90.00, 0.00)	0.251
≥10	-38.40 (-171.00, 0.00)	0.076	-33.00 (-130.00, -3.20)	0.133

Patients diagnosed with either moderate, high or very high risk of CKD at baseline were grouped and categorized according to the extent of weight loss achieved at 6 and 12 months after the start of semaglutide treatment. Then, the increment in UACR change with respect to baseline was calculated for each group. **P* value with respect to the group of CKD patients who lost <1% of their body weight at 6 months. †*P* value with respect to the group of CKD patients who lost <1% of their body weight at 12 months. Results are median (IQR). The one-tailed Mann-Whitney U test was used.

bl, baseline; CKD, chronic kidney disease; IQR, interquartile range; UACR, urine albumin to creatinine ratio.
