# Supplementary figures and images for: Influence of chronic kidney disease and its severity on the efficacy of semaglutide in type 2 diabetes patients: a multicenter real-world study
Source: Front Endocrinol (Lausanne). 2023 Oct 24;14:1240279. doi: 10.3389/fendo.2023.1240279 (PMC10634592; doi:10.3389/fendo.2023.1240279)

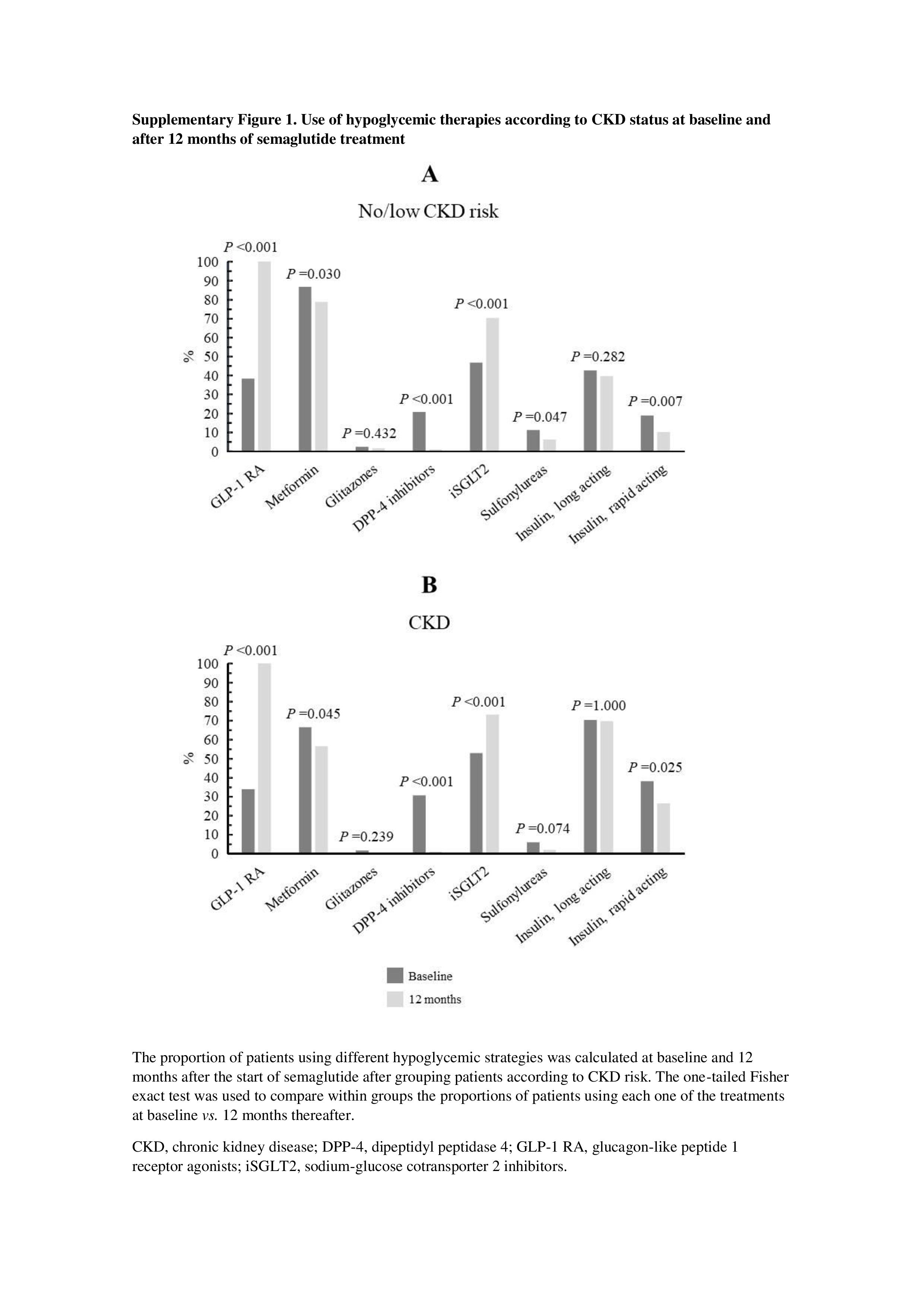

Supplement: Supplementary file 2 [file Image_1.jpg]

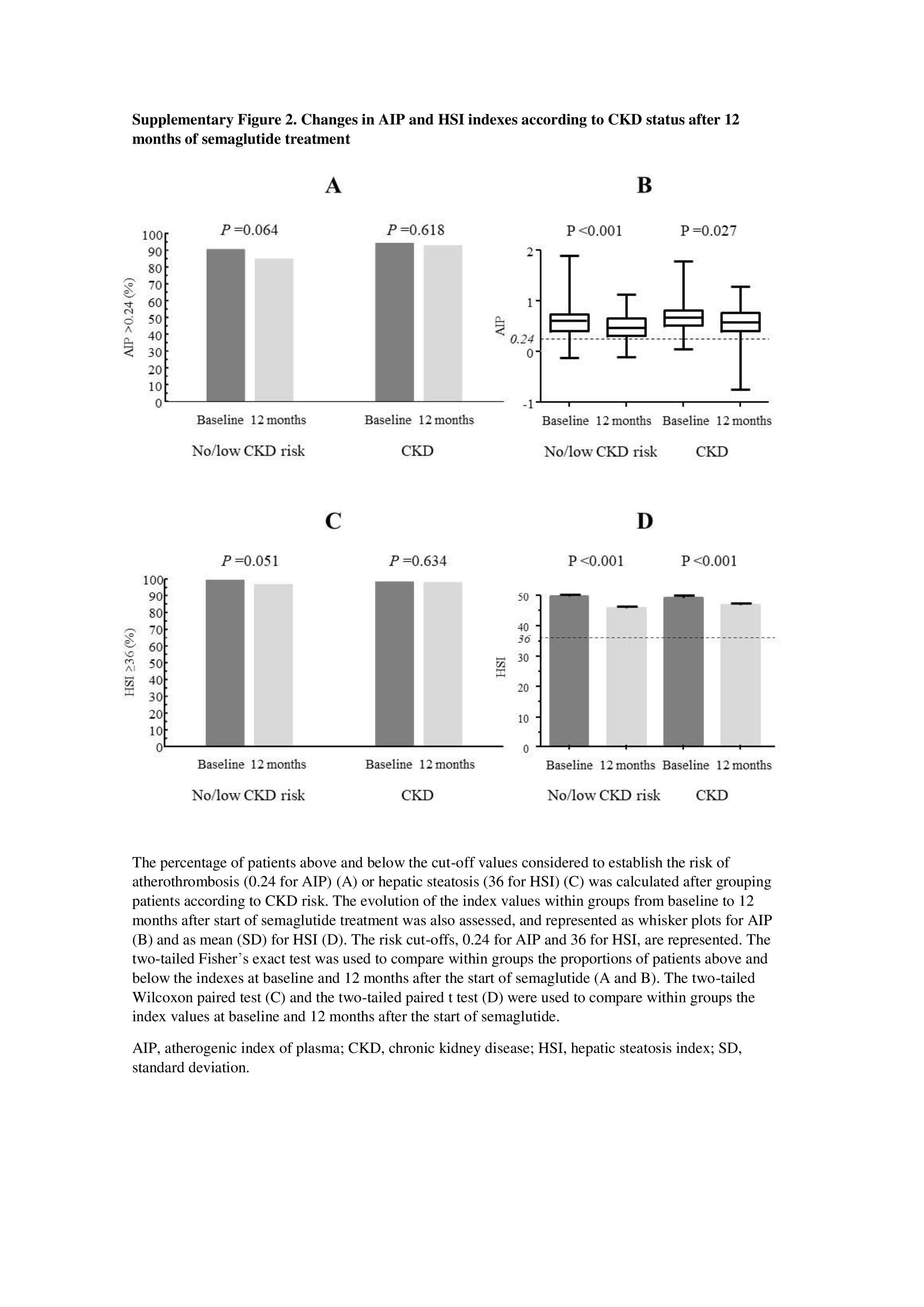

Supplement: Supplementary file 3 [file Image_2.jpg]
